# Validation of the Short Self-Regulation Questionnaire for Taiwanese College Students (TSSRQ)

**DOI:** 10.3389/fpsyg.2018.00259

**Published:** 2018-03-02

**Authors:** Yang-Hsueh Chen, Yu-Ju Lin

**Affiliations:** ^1^Institute of Teacher Education, National Chengchi University, Taipei, Taiwan; ^2^Library South, Center for Excellence in Teaching and Learning, Georgia State University, Atlanta, GA, United States

**Keywords:** self-regulation, factor analysis, validation study, psychometric, college students

## Abstract

While self-regulation has long been recognized as an important characteristic of an individual, instruments assessing the general aptitude of self-regulation remain limited especially in Asian countries. This study re-validated Carey et al.'s (2004) Short Self-Regulation Questionnaire based on a national sample of Taiwanese college students (*N* = 1,988). Item analysis, exploratory factor analysis, and confirmatory factor analysis (CFA) yielded 22 items in five internally consistent factors. Descriptive findings showed that, a lack of proactiveness and volitional control, and a decrease of self-regulation throughout the college span appeared to be an overarching problem among Taiwanese college students. Furthermore, male students achieved lower self-regulation scores than female ones, and students in Services and STEM-related majors are in the need of self-regulation enhancement. Due to the generic measurement of individual's self-regulation traits, the Taiwanese Short Self-regulation Questionnaire (TSSRQ) can be flexibly applied to various contexts and used to deal with different issues beyond learning such as college students' Internet or smartphone addiction. Through this study, we hope the validated TSSRQ can promote studies on self-regulation and associated antecedents and outcomes, in turn leveraging college students' life adjustment and well-being.

## Introduction

*Self-regulation (SR)* is an important capacity of a person to adapt to the variety of contextual circumstances that lead to healthy development of life (Zimmerman, 2002; Gestsdottir et al., 2010). Individuals with well-developed self-regulation know how to evaluate their own abilities, monitor their work progress, make efforts strategically, and utilize opportunities in the environment to help achieve their goals (Gestsdottir et al., 2010). Individuals with higher self-regulation were also found to achieve better psychological well-being in various contexts (e.g., Caprara and Steca, 2006; Allard, 2007). In educational contexts, *self-regulated learning (SRL)* denotes “an active, constructive process whereby learners set goals for their learning and attempt to monitor, regulate, and control their cognitions, motivation, and behavior, guided and constrained by their goals and the contextual features in the environment” (Pintrich, 2000, p. 453). While SR and SRL share the fundamental tenets but differ in scope, at times the two terms have been used interchangeably in the literature.

Over the decades, many SR/SRL models have been proposed, such as *self-regulated strategy development* (SRSD), (Graham et al., 1998) and *learning to learn model* (Hofer et al., 1998). Pintrich (2000, 2004) proposed a cognitive orientation SRL model that involves four phases of mental processes: (1) *Planning*, (2) *Monitoring*, (3) *Controlling*, and (4) *Reflecting*. Phase 1: *Planning* involves an activation of prior knowledge or metacognitive knowledge of goal setting for learning tasks; Phase 2: *Monitoring* refers to various monitoring processes of metacognitive awareness for changing learning tasks or contexts and self-needs; Phase 3: *Controlling* means individuals engage efforts to select and adapt cognitive strategies for learning or thinking, and Phase 4: *Reflecting*, which involves various reactions to make a self-examination and to evaluate learning tasks.

Likewise, Miller and Brown (1991) conceptualized a model of SR that includes seven phases: (1) *Receiving relevant informatio*n, (2) *Evaluating the information and comparing it to norms*, (3) *Triggering change*, (4) *Searching for options*, (5) *Formulating a plan*, (6) *Implementing the plan*, and (7) *Assessing the plan's effectiveness*. Overall, the above-mentioned SR and SRL models share the common assumptions that individuals are *active* and *constructive* agents to create their own thoughts and personal meanings; they are able to *observe, reflect, and adjust* their behaviors or inner thoughts. Individuals have their value systems to set goals and standards, and they evaluate their progress and achievement. Lastly, despite cultural and contextual influences, self-regulation plays a key role in mediating individual's cognition, motivation, and behaviors that lead to their achievement and performance (Pintrich, 2000; Boekaerts and Corno, 2005).

A number of instruments have been developed based on different models of SR/SRL, of which the most renowned is the *Motivated Strategies for Learning Questionnaire* (MSLQ) (Pintrich et al., 1991). The MSLQ details specific strategies and actions utilized by learners in a specific learning context (e.g., When reading for this course, I make up questions to help focus my reading). On the other hand, Brown et al. (1999) developed the Self-Regulation Questionnaire (SRQ) as the first attempt to measure the *general aptitude of self-regulation* (e.g., Once I have a goal, I can usually plan how to reach it) that applied to substance-abusing patients and a variety of other populations. More specifically, the SRQ was developed based on Miller and Brown (1991)'s seven-stage theorizing—each stage contains 9 items, totalling 63 items in the whole scale.

In a well-cited study, Carey et al. (2004) re-validated Brown's et al. (1999) SRQ with a sample of American college students (*N* = 391). A single-factor solution (31 items that covers all of the seven SR stages) emerged as a result, invariant across gender and semester. These items were then compiled into the short Self-regulation Questionnaire, abbreviated as SSRQ. Subsequent studies, including Neal and Carey (2005); Potgieter and Botha (2009); Vosloo et al. (2013), and Garzón Umerenkova et al. (2017) had re-validated the 31-item SSRQ with different samples and in different regions, but the dimensions and number of factors varied significantly across studies (see Table 1). It has been argued that the dimensions of self-regulation may vary by participants groups and culture (Vosloo et al., 2013; Garzón Umerenkova et al., 2017); accordingly, validation studies will be helpful to identify contextually specific dimensions to better capture self-regulation of a group of people in a given setting.

**Table 1:** Validation studies of SSRQ.

**Validation study**	**Neal and Carey, 2005**	**Potgieter and Botha, 2009**	**Vosloo et al., 2013**	**Garzón Umerenkova et al., 2017**
Sample/Context	*N* = 237	*N* = 385	*N* = 200	*N* = 831
	Undergraduate students taking the introductory psychology course	Undergraduate students taking the psychology course	Black African teachers in one of four education districts	Mainly undergraduate students from the field of Psychology, Primary Education and Teaching, and Science of Physical Activity and Sport. 290 participants are elementary school student teachers.
No. of Items	31 –>21	31 –>28	31 –>24	17
No. of Constructs	2	7	5	4
	1.Impulse control 2.Goal-setting	1.Monitoring 2.Decision Making 3.Learning from mistake 4.Perseverance 5.Self-evaluation 6.Creativity 7.Mindful awareness	1.Mindfulness 2.Self-efficacy 3.Monitoring change 4.Goal focus 5.Internal locus of control	1.Goal setting 2.Perseverance 3.Decision-making 4.Learning from mistake
Reliability (α)	0.84 (Impulse control) 0.86 (Goal-setting)	0.895(Overall)	0.86 (Overall) 0.80 (Mindfulness) 0.74 (Self-efficacy) 0.68 (Monitoring change) 0.63 (Goal focus) 0.63 (Internal locus of control)	0.87 (Overall) 0.81 (Goal setting) 0.71 (Perseverance) 0.76 (Decision-making) 0.79 (Learning from mistake)

A review of literature showed that, while to date a wealth of studies have been conducted under the umbrella of self-regulation, the majority of them focused on self-regulated learning (SRL) especially in school settings (e.g., Chen, 2012; Kao et al., 2013; Yeh et al., 2013; Huang and Chen, 2016). In Taiwan's higher education, Chen (2012) found that college students with greater SRL scores obtained better mid-term grades than those with lower SRL scores. Likewise, Kao et al. (2013) reported that college students with higher help-seeking (e.g., I will look for help if I cannot solve the problem) and self-control (e.g., I am responsible for my decision and action) capacities attained higher course grades. In terms of demographic influences, Yeh et al. (2013) reported that female college students obtained higher self-regulation scores than males in an online learning environment. Huang and Chen (2016) also found that female college students scored higher in perseverance and time management than their male counterparts. In addition, freshman and sophomore students tended to be more goal-oriented than senior students.

In contrast to SRL studies described right above, studies that investigate individual's general aptitude of self-regulation remain relatively small in number. What is more, a number of SRL instruments have been developed and validated across regions and groups, but the validation and application of the general SSRQ is still limited especially in Asian countries. Accordingly, the purpose of this study was to validate the SSRQ for Taiwanese college students (abbreviated as *TSSRQ*) in a larger scale. We hypothesize that the dimensions of TSSRQ will be different from other SSRQ conducted in other regions due to cultural differences, and we are specifically interested in the following two research questions:

RQ1: What are the dimensions of TSSRQ?

RQ2: What are Taiwanese college students' levels of self-regulation?

## Methodology

### Participants

The target population of this study is college students (aged between 18 and 22) in Taiwan. According to the census data by Taiwanese Ministry of Education, the total number of college students is approximately one million (1,015,398). Because Taiwan could be geographically divided into Northern, Central, Southern, and Eastern parts and the outlying islands, stratified sampling was applied to ensure that the participants came from all the five geographical areas. To further increase the representativeness of the sample, the researcher recruited participants from both public and private universities (see Table 2).

**Table 2:** Dispersion of sample across regions and sectors.

**Region**	**Public**	**Private**	**Total**
Northern (41%)	255	602	857
Middle (17%)	84	258	342
Southern (31%)	188	400	588
Eastern (7%)	33	104	137
Outlying Island (4%)	64	0	64
Total	624	1,364	1,988

We received 2,175 filled questionnaires. Any questionnaire with more than 5 missing values was deleted, and those with invariant answers (e.g., 1, 4, or 7 across all items) were removed from further analysis. In turn, 1,988 (91%) valid samples were retained, among them 945 were male (47.5%), 1,040 were female (52.3%) and 1 (0.1%) did not provide response of gender. More detailed demographic information is presented in Table 3.

**Table 3:** Demographic profiles of the participants.

		***N***	**%**
Gender	1. Male	945	47.5
	2. Female	1,042	52.4
	Unanswered	1	0.1
	Total	1,988	100.0
Grade level	1. Freshman	445	22.4
	2. Sophomore	590	29.7
	3. Junior	502	25.3
	4. Senior and above	439	22.0
	Unanswered	12	0.6
	Total	1,988	100.0
Study major	1. Education	188	9.5
	2. Humanities and arts	401	20.2
	3. Social sciences, business and law	361	18.2
	4. Science	156	7.8
	5. Engineering, manufacturing and construction	399	20.1
	6. Agriculture	21	1.1
	7. Health and welfare	110	5.5
	8. Services	111	5.6
	9. Other/miscellaneous	204	10.3
	Unanswered	37	1.9
	Total	1,988	100.0

### Instrument and procedure

In this study we adopted Carey et al.'s (2004) 31-item SSRQ as the original item pool. This makes our study more comparable to prior validation studies such as Potgieter and Botha (2009); Vosloo et al. (2013); Garzón Umerenkova et al. (2017), and Neal and Carey (2005). Moreover, this short version is more practical/flexible to be used in survey studies that contain multiple scales. We translated the items into Chinese, and then the translated items were reviewed by two scholars with backgrounds of Educational Psychology to ensure that the translations adhere to the meaning of the original English version. In addition, two undergraduate research assistants helped check the Chinese items to make sure that college students could understand the item expressions well.

Regarding data collection, an ethics approval was not required as per institutional and national guidelines and regulations. In addition, it was entirely voluntary for students to participate the anonymous survey, and consent was obtained upon the survey completion. Due to the fact that college students' study majors included nine main domains (see Table 3), we deemed it appropriate and efficient to find the courses that include students from diverse academic backgrounds and study majors. Therefore, we contacted 13 general education and teacher education centers in selected universities and obtained their permissions to help administer the survey. Bundled survey questionnaires were mailed to the program administrators or directly to the instructors. Then they brought the questionnaires to the class and explained the purpose of the study. Students who agreed to participate went on to complete the anonymous survey, while those who were unwilling to participate could leave it blank without any forms of penalty. After students completed the questionnaires, the administrators and instructors mailed them back to the researchers. The data collection lasted for 3 months.

### Data analysis

In order to answer Research Question 1, “What are the dimensions of TSSRQ?” we proceeded with item analysis, exploratory factor analysis (EFA), confirmatory factor analysis (CFA), and reliability tests. More specifically, missing values were imputed with dimension means. All the 31 SSRQ items were scrutinized for their mean, variance, and skewness scores, and students within high and low groups were compared at item level in order to remove low quality items.

Prior to EFA, we used SPSS 22.0 to randomly select one third of the sample (*N* = 558) for EFA. Also, Bartlett sphericity test and the KMO index were calculated via SPSS 22.0 to determine the suitability of factor analysis. The principal axis factoring method with oblimim rotation was used in our EFA, and the Eigenvalue of 1.00 was set at as the threshold to determine the number of factors/dimensions. The threshold factor loading of 0.40 was used to maintain or remove items. The remaining two-thirds of the sample (*N* = 1,330) were used in our CFA to verify the dimensions generated by EFA. We used the Amos 21 (Arbuckle, 2012) program, and Maximum Likelihood estimation was applied. Model fit was evaluated using the Chi-square fit index, the comparative fit index (CFI), the root mean square error of approximation (RMSEA), and the standardized root mean square residual (SRMR) (Jackson et al., 2009). Lastly, Cronbach's alphas of the subscales and the total scale were calculated with SPSS 22.0 to determine the internal consistency of TSSRQ.

In order to answer Research Question 2, “What are Taiwanese college students' levels of self-regulation?” descriptive statistics were applied to demonstrate participant's mean scores on the TSSRQ dimensions. Furthermore, independent sample *t*-test and one-way ANOVA were utilized to detect any significant differences between demographic variables, including gender, grade level, and study major on the TSSRQ total score.

## Results

### Validation of SSRQ dimensions

#### Item analysis

Descriptive analysis results showed that, the mean scores of each item lay between 3.48 and 5.23, and the standard deviations were all above 1.00. Moreover, the maximum value of the skewness was 0.693 in absolute value, indicating good dispersion of scores across all the 31 items. In addition, participants were sorted into “high” and “low” groups based on the 23 and 77 percentile ranks of their TSSRQ total scores. Independent sample *t*-test results showed that, for each of the 31 items, the high and low group differed significantly at 0.001 level, indicating good item discrimination. Lastly, we calculated the alpha values for each item in relation to the total scale. Two items, “(SSRQ22) When it comes to deciding about a change, I feel overwhelmed by the choices” and “(SSRQ31) It's hard for me to notice when I've had enough (alcohol, food, sweets)” revealed an increase of the overall Cronbach alpha if these items were deleted. Based on the above information, we removed SSRQ22 and SSRQ31 from further data analysis, and the number of items became 29 in number.

#### Exploratory factor analysis (EFA)

The Bartlett sphericity test (*X*^2^ = 25,724, *df* = 406) and the KMO index (0.942) provided evidence the data were appropriate for factor analysis. In the first round of EFA we yielded five factors in which two *Planning/Goal Setting* items: “(SSRQ01) I have trouble making plans to help me reach goals” and “(SSRQ02) I have a hard time setting goals for myself” grouped together as a single factor. Four items, namely items SSRQ18, 24, 8, and 20 had to be eliminated because their factor loadings did not reach the threshold of 0.40.

We removed items 18, 24, 8, and 20 and conducted the second round of EFA. Again we yielded five factors, and again the two Goal Setting items grouped together in a single factor. Now item 12 needed to be eliminated because it had high cross-loadings on two factors; item 25 should be deleted because its factor loading did not reach 0.40. Also we determined to remove item 27 because the narration was very different from the other three items, making it hard to explain the whole factor.

Upon deleting SSRQ12, 25, and 27 we conducted the third round of EFA. Surprisingly this time we only yielded four factors in which the original two Goal Setting items merged into the Mindfulness factor. Considering that Planning/Goal setting is an important step/construct of self-regulation (Pintrich, 2000, 2004; Zimmerman, 2002), also the two Goal Setting items had repeatedly grouped together in the previous EFAs, we did not immediately remove these two items from the Mindfulness factor. Rather, we used the Mplus program to test/compare models that contained 2, 3, 4, 5, and 6 factors. Results indicated that the five-factor solution yielded the best model fit (*X*^2^ = 337.381, *df* = 144, *p* = 0.0000, CFI = 0.966, RMSEA = 0.050, SRMR = 0.022) as compared to the four-factor model (*X*^2^ = 575.124, *df* = 126, *p* = 0.0000, CFI = 0.931, RMSEA = 0.067, SRMR = 0.028). Therefore, we accepted the five-factor solution and then changed the criteria from Eigen value >1 to directly setting 5 factors in our latest (fourth round) EFA with SPSS 22.0. This time the two Goal Setting items (SSRQ01 and SSRQ01) grouped together again, and no more items needed to be eliminated (see Table 4). The Eigen values for the five factors were 7.518, 2.950, 1.484, 1.167, and 0.866 respectively, and together the five factors explained 53.65% of the total variance. More details of the factors are described below:

**Factor 1–*Goal Attainment* (GA):** This factor contains 7 items that present Taiwanese college students' actions to keep track of their progress to reach their goals. A sample questions is “(SSRQ06) When I'm trying to change something, I pay attention to how I'm doing.” All of the items are positively formulated.
**Factor 2–*Mindfulness* (MF):** This factor includes 7 items that assess the participant's mindful awareness and volition to stick to their goals. Sample questions are: (SSRQ16) Most of the time I don't pay attention to what I'm doing. (R) and (SSRQ11) I have trouble following through with things once I've made up my mind to do something.
**Factor 3–*Adjustment* (AD):** This factor contains 3 items to portray individual's ability to make changes according to the mistakes they make or challenges they face. A sample question is “(SSRQ21) As soon as I see a problem or challenge, I start looking for possible solutions.” Among the three items, the first item (SSRQ17) was negatively formulated.
**Factor 4–*Proactivenss* (PA):** This factor contains 3 items that reflect a person's vigorous actions to learn quickly from mistakes, stick to a good plan, and actively seek possibilities to change something. For instance, “(SSRQ30) I can usually find several different possibilities when I want to change something”. All the three items are positively formulated.
**Factor 5–*Goal Setting* (GS):** This factor contains 2 items that assess Taiwanese college students' ability to plan and set clear goals. A sample question is, “(SSRQ02) I have a hard time setting goals for myself”; both of the items are negatively formulated.

**Table 4:** Factor structure and item loadings of TSSRQ.

	**Goal Attainment**	**Mindfulness**	**Adjustment**	**Proactiveness**	**Goal Setting**
	**(GA)**	**(MF)**	**(AD)**	**(PA)**	**(GS)**
6. (SSRQ06) When I'm trying to change something, I pay attention to how I'm doing.	0.784				
5. (SSRQ05) I set goals for myself and keep track of my progress.	0.771				
3. (SSRQ03) Once I have a goal, I can usually plan how to reach it.	0.614				
13. (SSRQ13) I'm able to accomplish goals I set for myself.	0.532				
14. (SSRQ14) If I make a resolution to change something, I pay a lot of attention to how I'm doing.	0.512				
19. (SSRQ19) I usually keep track of my progress toward my goals.	0.490				
9. (SSRQ09) I have personal standards, and try to live up to them.	0.486				
10. (SSRQ10) I get easily distracted from my plans. (R)		0.722			
11. (SSRQ11) I have trouble following through with things once I've made up my mind to do something. (R)		0.675			
15. (SSRQ15) I put off making decisions. (R)		0.666			
4. (SSRQ04) I give up quickly. (R)		0.553			
7. (SSRQ07) I don't notice the effects of my actions until it's too late. (R)		0.509			
16. (SSRQ16) Most of the time I don't pay attention to what I'm doing. (R)		0.481			
26. (SSRQ26) I have trouble making up my mind about things. (R)		0.437			
17. (SSRQ17) I don't seem to learn from my mistakes. (R)			0.585		
23. (SSRQ23) I learn from my mistakes.			0.506		
21. (SSRQ21) As soon as I see a problem or challenge, I start looking for possible solutions.			0.455		
29. (SSRQ29) I can stick to a plan that is working well.				0.723	
28. (SSRQ28) I usually only have to make a mistake one time in order to learn from it.				0.708	
30. (SSRQ30) I can usually find several different possibilities when I want to change something.				0.668	
1. (SSRQ01) I have trouble making plans to help me reach goals. (R)					0.928
2. (SSRQ02) I have a hard time setting goals for myself. (R)					0.761

#### Confirmatory factory analysis (CFA)

**Figure 1:**
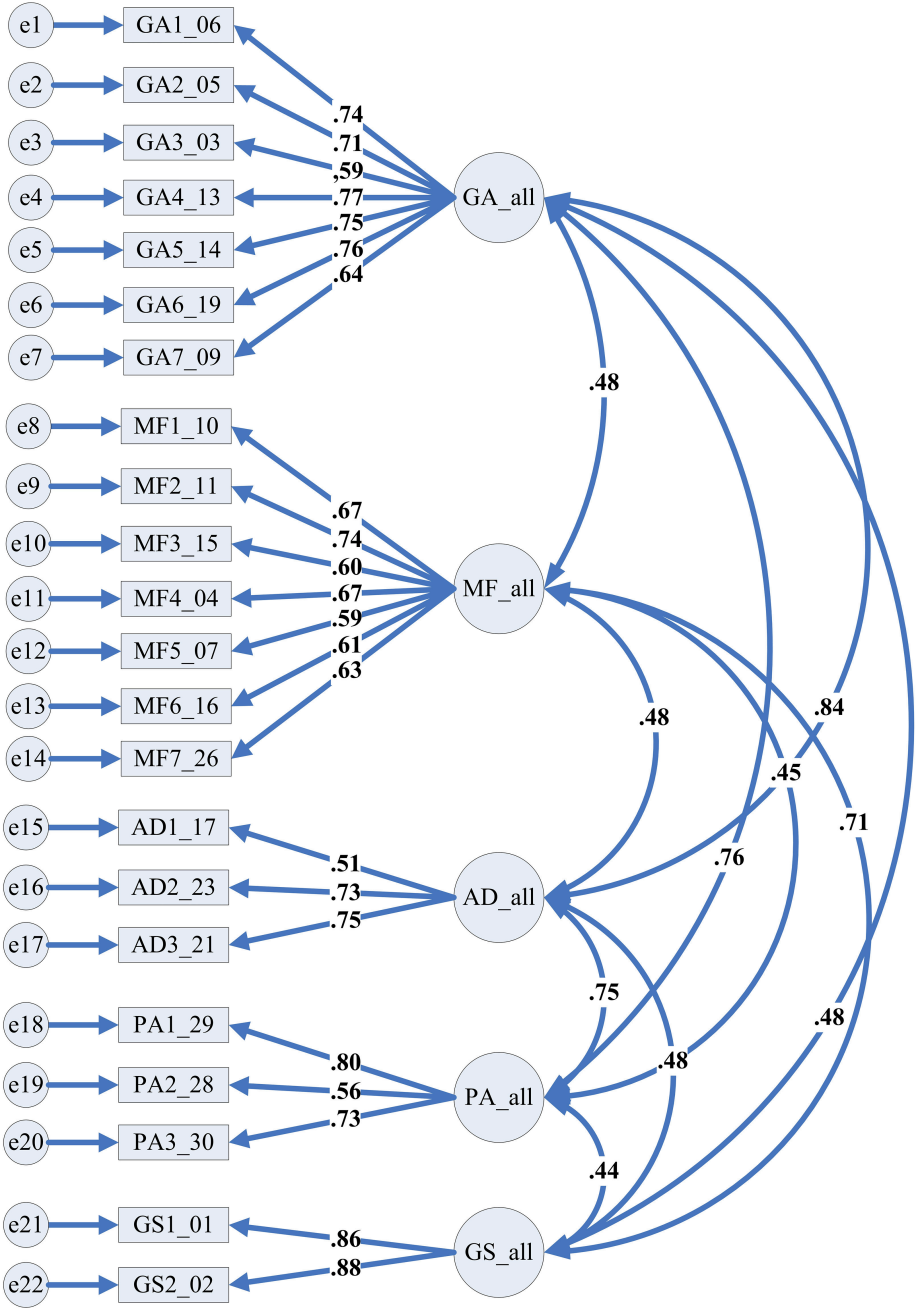
Path model and factor loadings of CFA.

Following the above EFA results we constructed a model that included the 5 factors measured by the 22 items. These factors included 2 to 7 items with the factor loadings ranging between 0.51 and 0.88. The correlations among factors ranged from 0.44 to 0.84. Model fit results showed that Chi-square was significant (*X*^2^ = 1540.89, *df* = 199, *p* = 0.00), and the values of CFI (0.90), RMSEA (0.07), and SRMR (0.06) were all within the proper range. Overall, we deemed the model fit acceptable without any change from modification indices. Figure 1 displays the path model and the factor loadings within each latent factor. Note that the factor loadings of the items are all greater than 0.50 and can be considered important for the associated dimensions.

#### Reliability test

As shown in Table 5, the results ranged from 0.803 to 0.875, indicating satisfactory internal consistency.

**Table 5:** Dimensions, numbers of items, and internal consistency of the revised SSRQ.

**Dimension**	**Items**	**Cronbach's α**
Goal Attainment (GA)	7	0.875
Mindfulness (MF)	7	0.855
Adjustment (AD)	3	0.835
Proactiveness (PA)	3	0.803
Goal Setting (GS)	2	0.818
Total	22	0.908

### Taiwanese college students' self-regulation

Table 6 presents the profiles of Taiwanese college students' self-regulation. Overall, the average score of the total scale lay at 4.73, moderately above the mid-point of the seven scale points. Paired-sample *t*-test were applied to detect mean differences between the five SSRQ dimensions. Results showed that all the paired-sample *t*-test were significant, verifying that the participant's dimension scores, from highest to lowest are: (1) *Adjustment*, (2) *Goal Attainment*, (3) *Goal Setting*, (4) *Proactiveness*, and (5) *Mindfulness*. It is noticeable that *Goal Setting* has the standard deviation of 1.37, which is highest among the five dimensions.

**Table 6:** Taiwanese college students' self-regulation profiles.

**Dimension**	***M***	***SD***	**Ranking**
Goal Attainment (GA)	4.88	0.91	AD>GA>GS>PA>MF
Mindfulness (MF)	4.31	1.00	
Adjustment (AD)	5.13	0.96	
Proactivenss (PA)	4.60	0.96	
Goal Setting (GS)	4.71	1.37	
TSSRQ-Total	4.73	0.78	

We further explored demographic differences in the TSSRQ total score by gender, grade level, and study major through ANOVAs. Levene tests were all insignificant, indicating homogenous variances across groups. Results showed that females achieved higher self-regulated scores than males [*df*_(1985)_, *t* = −2.49, *p* = 0.013^*^, η^2^ = 0.002]. In terms of grade level, significant differences were detected in the TSSRQ total score [*df*_(4, 1971)_, *F* = 4.38, *p* = 0.002^**^, η^2^ = 0.007]. *Post-hoc* analysis (using the Bonferroni method) indicated that freshmen and sophomores achieved higher self-regulation scores than seniors and above. As with study major, overall the ANOVA result was significant [*df*_(8, 1942)_, *F* = 4.78, *p* = 0.000^***^, η^2^ = 0.013]. *Post-hoc* Bonferroni analysis indicated that, overall students majoring in *Health and Welfare* and *Education* scored highest among the nine categories of study major. By contrast, students who majored in *Services* scored lowest in self-regulation. More details of the comparison results are presented in Tables 7–9.

**Table 7:** Independent *t*-test of TSSRQ total score with gender and one-way analyses of TSSRQ with grade level and study major.

	***M* (*SD*)**	***df***	***t/F***	***p***	**η^2^**
Gender	4.73 (0.78)	(1985)	−2.49	0.013^*^	0.002
Grade level	4.73 (0.78)	(4, 1971)	4.38	0.002^**^	0.007
Major	4.72 (0.79)	(8, 1942)	4.78	0.000^***^	0.013

* *Coefficient is significant at the 0.05 level (2-tailed)*.

** *Coefficient is significant at the 0.01 level (2-tailed)*.

*** *Coefficient is significant at the 0.001 level (2-tailed)*.

**Table 8:** *Post-hoc* (Bonferroni) analyses among gender and grade levels.

**Gender**		**Grade level**			
***M_*female*_* (*SD*)**	***M_*male*_* (*SD*)**	***M_*freshman*_* (*SD*)**	***M_*sophomor*_* (*SD*)**	***M_*junior*_* (*SD*)**	***M_*senior*_* (*SD*)**
4.77 (0.77)	4.68 (0.79)	4.78 (0.80)	4.76 (0.79)	4.73 (0.76)	4.60 (0.73)
female > male^*^		freshman > senior and above^**^ sophomore > senior and above^*^			

* *Coefficient is significant at the 0.05 level (2-tailed)*.

** *Coefficient is significant at the 0.01 level (2-tailed)*.

**Table 9:** *Post-hoc* (Bonferroni) analyses among majors.

**Major**	***n***	***M***	***SD***	***Post-hoc***
Education (1)	188	4.88	0.81	1>5^*^, 1>8^*^, 1>9^*^, 7>4^*^, 7>5^**^, 7>8^*^, 7>9^**^
Humanities and arts (2)	401	4.75	0.77	
Social sciences, business and law (3)	361	4.81	0.77	
Science (4)	156	4.63	0.73	
Engineering, manufacturing and construction (5)	399	4.65	0.78	
Agriculture (6)	21	4.65	0.96	
Health and welfare (7)	110	4.96	0.85	
Services (8)	111	4.59	0.69	
Other/Miscellaneous (9)	204	4.60	0.74	

* *Coefficient is significant at the 0.05 level (2-tailed)*.

** *Coefficient is significant at the 0.01 level (2-tailed)*.

## Discussion

### Dimensions of TSSRQ

In this study we yielded five factors of self-regulation, namely *Goal Setting, Goal Attainment, Mindfulness, Adjustment, and Proactiveness* based on a national sample of Taiwanese college students. We found that the former four factors (i.e., *Goal Setting, Goal Attainment, Mindfulness, Adjustment*) are closely related to the dimensions obtained in prior validation studies. For *Goal Setting*, Neal and Carey (2005); Pichardo et al. (2014), and Garzón Umerenkova et al. (2017) all discovered this same factor. The items such as “I have trouble making plans to help me reach goals” and “I have a hard time setting goals for myself” are commonly included under the construct of *Goal Setting*. On the other hand, our *Goal Attainment* aligns with *Goal Orientation* in Gavora et al. (2015) study and *Internal Locus of Control* by Vosloo et al. (2013). Common items under the construct of *Goal Attainment* include “I have personal standards, and try to live up to them” and “I'm able to accomplish goals I set for myself.”

*Mindfulness* has been identified as an important factor across SSRQ validation studies; for instance, Potgieter and Botha (2009) and Vosloo et al. (2013) respectively included *Mindfulness* and *Mindful Awareness* dimensions in their SSRQs. Common items include “I don't notice the effects of my actions until it's too late” and “I have trouble following through with things once I've made up my mind to do something”. While under a quite different name, *Adjustment* in this study resembles *Learning from Mistakes* coined by Potgieter and Botha (2009); Pichardo et al. (2014), and Garzón Umerenkova et al. (2017). Adjustment was also similar to *Self-direction* in the SRQ validation study by Gavora et al. (2015). Common items across studies include “I don't seem to learn from my mistakes” and I learn from my mistakes. The above construct alignment between the current study and prior studies provide evidence that our TSSRQ should bear good construct and criterion-related validities.

Yet, in this study *Proactiveness* has been perceived by Taiwanese college students as a unique factor of self-regulation. We suspect that it has been influenced by the general conception in Taiwan that college students lack active attitudes and momentum, as frequently reported in news media and also perceived by American EFL teachers in Lin's et al. (n.d.) study. In a recent report on Taiwanese college students' self-directed learning readiness (*N* = 1,049), Cheng and Cheng (2014) also found that students' *Active Learning* scored lowest among the 10 scale dimensions. The uniqueness of the Proactivenss dimension provides preliminary support of our hypothesis that the dimensions of TSSRQ will be different from other SSRQs due to cultural differences. Future studies are suggested to investigate the role of proactiveness in Taiwanese college students' academic and daily lives.

### Taiwanese college students' levels of self-regulation

In this study our participants scored highest on *Adjustment* (*M* = 5.13), which means that Taiwanese college students were apt to make changes based on prior mistakes; also they think about solutions upon encountering problems. This result is consistent with Potgieter and Botha's (2009)'s study wherein South African college students scored higher in the *Learning from Mistake* dimension. One possible explanation of such consistency is that college students are relatively young and might be more flexible or malleable to adjust themselves (Wilson, 2008). On the other hand, Taiwanese college students scored lowest on *Mindfulness* (*M* = 4.31), followed by *Proactiveness* (*M* = 4.60). This means that in addition to the aforementioned problem of passivity, Taiwanese college students also suffer from poor conscious awareness, and worse, weak resolution/perseverance to follow through their plans. Such a lack of volitional control has also been reported by Kao et al. (2013) study wherein Taiwanese college nursing students (*N* = 537) scored lowest on *Self-management* among other dimensions of self-directed learning. It is interesting that the standard deviation of *Goal Setting* (*SD* = 1.37) was highest among all the studied variables, meaning that some students are good at planning and settings goals but others may not. It would be helpful that college instructors or mentors help students reflect on their personal aspirations, and work with them to set appropriate goals for study as well as personal lives and future growth.

Our follow-up comparisons indicated that females scored higher in self-regulation total score than male students. This result is in line with Yeh et al. (2013) national study that Taiwanese female college students applied more self-regulated learning strategies in the e-learning environment than their male counterparts. Yet, Shimai et al. (2006) found that Japanese male college students achieved better self-regulated scores than females (as measured by the VIA Inventory of Strengths, Peterson and Seligman, 2004), which is inconsistent with the present study.

Regarding grade level, seniors and above were found to be the least self-regulated group. In fact, a glance on the participant's self-regulation scores showed a gradual decrease from freshman to senior and above. The above results are inconsistent with prior SR/SRL studies on Taiwanese college students, for example, Chen (2012) found no significant difference on the use of self-regulation strategies among Taiwanese college students majoring in early childhood education at different grade levels. By contrast, Kao et al. (2013) reported that Taiwanese college nursing students aged between 20 (junior) and 21 (senior) were more self-regulated than students aged between 18 (freshman) and 19 (sophomore). Considering that the effect size is very small in our study (η^2^ = 0.007), we recommend more nationwide studies to test/cross-validate our findings; also it would be interesting to examine whether trainings in different study majors would possibly influence students' self-regulation at different grade levels.

While the effect size is small, this study found that in general, students majoring in (1) *Health and Welfare*, and (2) *Education* were statistically more self-regulated than those from the other study majors. For the domain of health and welfare, it has long been recognized that medical education requires long and rigorous training, and a wealth of studies have documented the workload and stress by medical students at college level (e.g., Radcliffe and Lester, 2003; Dahlin et al., 2005). For instance, students in Dahlin et al.'s (2005) study expressed that preparing for examinations and acquiring professional knowledge, and transitions such as between school and medical school, and from clinical training to approaching qualification were particularly stressful. It follows that, students under such workloads and requirements may need to develop higher self-regulation and associated strategies to survive.

In the area of *Education*, during their course of study students are immersed in educational psychology, various learning theories and pedagogies, and they experience lesson planning and trial teaching in practicum courses. Such professional training with embedded SR cultivation may be helpful for students to internalize self-regulation into their mindsets, in turn leveraging their TSSRQ scores. Surprisingly, students majoring in STEM-related domains (i.e., *Category 4*: *Science* and *Category 5*: *Engineering, manufacturing and construction*, see Table 3) were among the lowest self-regulation groups. A glance of self-regulation means scores (see Table 9) showed the data for the two domains were 4.63 and 4.65 respectively, which are far below the aforementioned *Health and Welfare* (*M* = 4.96), and *Education* (*M* = 4.88). Following Miller's (2015) argument that self-regulation is highly desirable for STEM students to apply and synthesize the fundamental disciplinary concepts, it is important that more research efforts to be dedicated to examining current curriculum and instruction in STEM related programs in Taiwan, and devise strategies to promote student self-regulation and high-ordered learning outcomes.

## Limitations, implications, and future directions

Despite that we have yielded a reasonable composite of SR dimensions, only two or three items are included in *Goal Setting, Adjustment*, and *Proactiveness* dimensions due to the original small item pool (i.e., 31 items in SSRQ) and the removal of 9 items during item analysis and EFAs. In the future, more items, especially locally customized items are suggested to add to these dimensions to further strengthen the psychometric quality of TSSRQ. Furthermore, this TSSRQ is self-report in nature, and inevitably it is under certain threat of social desirability bias (Fisher, 1993). As such, interpretation of TSSRQ results should proceed with caution. In some cases, observations, interviews, or focus groups can be applied to supplement scale data and gain more holistic perspectives of self-regulation among college students.

Another salient limitation is that although statistically significant differences were found between gender, grade levels, and study majors, the effect sizes were actually very small, limiting practical significance of data. Therefore, readers should be cautious not to over-interpret the statistical results. Instead, we hope this study could help raise our awareness about potential demographic influences on college students' self-regulation. On the other hand, more research efforts can be dedicated to investigating college students' voices across gender, grade levels, and study majors. Also it would be a worthwhile endeavor to explore factors that may potentially mediate or moderate the influences of demographics on college students' self-regulation.

In this study we validated the short self-regulation scale for Taiwanese college students (TSSRQ). Five dimensions of *Goal Setting, Goal Attainment, Mindfulness, Adjustment*, and *Proactiveness* were obtained. Since TSSRQ measures individual's general traits of self-regulation, the instrument can be flexibly applied to various contexts and issues. For example, researchers can explore the extent to which self-regulation is associated with Internet or smartphone addiction among college students; also it can be applied to examine whether SR can predict college students' psychological well-being and life adjustment. Aside from research, the TSSRQ can be used as a checklist for college students to reflect on their self-regulated thoughts, actions, and habits at school and in their daily lives. For teachers, students' TSSRQ data can be referenced to understand their strengths, weaknesses, and readiness for self-regulated learning, in turn devise creative instructional strategies and activities (e.g., team-based problem solving, peer coaching, or learning from failure/mistakes activities) that cultivate self-regulation, exploit inner potentials, and inspire positive aspirations/volitions of the students and the teachers as well (Seligman and Csikszentmihalyi, 2000).

## Conclusion

This study purports to validate the SSRQ for Taiwanese college students in a larger scale. The revised TSSRQ include dimensions of *Goal Setting, Goal Attainment, Mindfulness, Adjustment*, and *Proactiveness*. We consider these dimensions to be both encompassing and parsimonious to portray Taiwanese college students' self-regulation. This study also echoes Gavora's (2015) argument that SRQ does not follow Miller and Brown's (1991) seven-step theorizing but is more reflective of aspects of personal traits. The present study also aims to examine Taiwanese college students' levels of self-regulation. We found that the lack of proactiveness and volitional control, and a decrease of self-regulation throughout the college span appeared to be an overarching problem among Taiwanese college students. Furthermore, male students were less self-regulated, and students in Services and STEM-related majors appeared to be in need of SR enhancement. While a detailed discussion of intervention strategies is beyond the scope of this study, here we suggest that future studies keep examining the identified problems, and—perhaps consulting Boekaerts and Corno's (2005) categories of intervention and Deci and Ryan's (1985, 2002) autonomy support strategies—continue to devise, customize, and test self-regulation interventions to support college students' autonomous learning, life adjustment, and well-being throughout their college span.

## Ethics statement

This study was exempt from ethical approval procedures because it only involves the survey procedures without information which can be identified, directly or through identifiers linked to the human subjects.

## Author contributions

Y-HC: Serves as the first author to design the entire research study, collect and analyze data, and complete the manuscript; Y-JL: Works as the second author to assist in data analysis and to complete the manuscript.

### Conflict of interest statement

The authors declare that the research was conducted in the absence of any commercial or financial relationships that could be construed as a potential conflict of interest.
